# Determination of Peak Purity in HPLC by Coupling Coulometric Array Detection and Two-Dimensional Correlation Analysis

**DOI:** 10.3390/s22051794

**Published:** 2022-02-24

**Authors:** Julio Enrique Oney-Montalvo, Ksenia Morozova, Manuel Octavio Ramírez-Sucre, Matteo Scampicchio, Ingrid Mayanin Rodríguez-Buenfil

**Affiliations:** 1 Centro de Investigación y Asistencia en Tecnología y Diseño del Estado de Jalisco, A.C. Sede Sureste, Tablaje Catastral No. 31264, km 5.5 Carretera Sierra Papacal-Chuburna Puerto, Interior del Parque Científico y Tecnológico Yucatán, Mérida 97302, Yucatán, Mexico; juoney_al@ciatej.edu.mx (J.E.O.-M.); oramirez@ciatej.mx (M.O.R.-S.); irodriguez@ciatej.mx (I.M.R.-B.); 2 Faculty of Science and Technology, Free University of Bozen-Bolzano, Piazza Università 5, 39100 Bolzano, Italy; ksenia.morozova@unibz.it

**Keywords:** coulometric sensor, electrochemical sensor, 2D correlation analysis, food sensors, liquid chromatography, mass spectrometry

## Abstract

This work aims to evaluate the purity of chromatographic peaks by a two-dimensional correlation (2D-corr) analysis. Such an analysis leads to two contour plots: synchronous and asynchronous. The synchronous contour plot provides information on the number of peaks present in the chromatogram. The asynchronous contour plot reveals the presence of overlapping species on each peak. The utility of 2D-corr analysis was demonstrated by the chromatographic analysis of *Capsicum* chili extracts obtained by HPLC coupled with a coulometric array of sixteen detectors. Thanks to 16 electrochemical sensors, each poised at increasing potentials, the resulting 2D-corr analysis revealed the presence of at least three species on the peak located at a retention time of 0.93 min. Mass spectrometry (MS) analysis was used to analyze the coeluting species, which were identified as: quinic acid (3.593 min), ascorbic acid (3.943 min), and phenylalanine (4.229 min). Overall, this work supports the use of 2D-corr analysis to reveal the presence of overlapping compounds and, thus, verify the signal purity of chromatographic peaks.

## 1. Introduction

Analytical interferences occur in chromatography when the signal of a substance is coeluting with the signal of the analyte of interest. The presence of interferences is a common source of errors during analytical quantification [1]. Such errors are especially critical for governmental authorities that, through their analysis, must guarantee the quality and safety of foods, i.e., protecting consumers against adulterations and contaminations [2]. One clear example is the contamination of Habanero peppers by mycotoxins, pesticides, or heavy metals, as well as their adulteration with dyes and foreign matter [3]. In such cases, the effect of concomitant species in the analytical signal causes a systematic error on the analytical result, i.e., compromising the accuracy of the final outcome.

To verify the presence of interferences in chromatographic peaks, it is generally recommended to proceed with a series of preliminary checks. One is certainly the injection of the diluent blank [4]. Afterwards, it is possible to test blank samples spiked with known impurities and check their retention times [4]. Finally, when real samples are analyzed, a simple visual analysis of the resulting peak shapes may reveal a problem of symmetry or tailing, which may be associated with interference problems. In the case of the PDA detector, the most common method to analyze peak purity is comparing spectra within a peak—a pure peak has matching spectra throughout the chromatogram [5]. However, the final decision seems rather subjective and time-consuming. Recent attempts to identify the presence of interferences have been proposed by the application of principal component analysis (PCA), which has been applied in chromatographic purity analysis to monitor subtle changes in the chromatographic pattern [6]. Although PCA was proven to be a robust and reliable tool to evaluate the purity of the peaks, it showed the disadvantage of not being able to automatize the analysis. The PCA analysis requires qualified personnel with chemometric knowledge to verify the presence of interferences [6,7]. This has led to the search for alternatives, such as 2D-corr analysis.

Recently, 2D-corr analysis has been applied mainly in spectroscopy to analyze the changes occurring in spectral signals (e.g., IR, UV) under a specific physical perturbation (e.g., electrical, chemical, thermal, mechanical, optical) [8].

**Figure 1:**
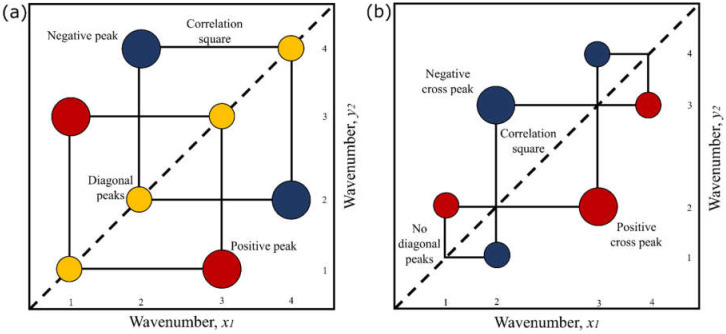
Schematic contour map of: (**a**) synchronous 2D-corr spectrum, (**b**) asynchronous 2D-corr spectrum. 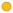 Auto-peaks located along the main diagonal. 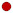 Peaks with a positive sign. 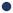 Peaks with a negative sign.

The analysis is based on the Fourier transform of the original dynamic spectra, leading to real and imaginary cross-correlation functions, which are called, respectively, synchronous or asynchronous correlation spectra maps [9]. Synchronous 2D-corr spectra correspond to the real part of the cross-correlation function and represent simultaneous changes of the measured spectral intensity variations induced by a perturbation [10]. This map (Figure 1a) is confirmed by auto-peaks located along the main diagonal and cross-peaks located at the off-diagonal positions. Cross-peaks with coordinates A and C have negative signs. This indicates that the intensity in one band is increasing, while the other is decreasing. On the other hand, cross-peaks with coordinates B and D have a positive sign, where both bands decrease or increase together.

In contrast, asynchronous 2D-corr spectra represent sequential or successive changes of spectral intensities induced by perturbations [11]. This map (Figure 1b) consists of only cross-peaks located at the off-diagonal positions. In this example, correlation band pairs are observed: A–B, A–D, B–C, and C–D, which conform to four correlation squares [11]. A cross-peak only appears in an asynchronous map when the intensities of two spectral features change out of phase with each other, which makes them asymmetric with respect to the diagonal line [11].

Two-dimensional correlation analysis (synchronous and asynchronous) has been successfully applied to evaluate the data obtained by several analytical techniques, such as IR, Raman, NMR, UV/VIS spectroscopy, and mass spectrometry [12,13,14,15,16]. Previous research has showed that the information obtained by 2D-corr analysis can be used to analyze overlapped peaks, which cannot be separated with a one-dimensional (1D) signal [17]. From the interpretation of synchronous and asynchronous spectra, it is possible to obtain valuable information that could be applied to the analysis of chromatographic signals. However, to the best of our knowledge, no studies have shown the application of 2D-corr for analyzing the signal of the coulometric array detector (CoulArray).

For this reason, the main aim of this work is to apply 2D-corr analysis to detect impurities in peaks obtained with a CoulArray detector. This detector consists of serial cell blocks containing porous graphite working electrodes, each poised at different working potentials versus a common palladium reference electrode, offering sensitivity substantially higher than UV or fluorescence detectors [18,19]. To demonstrate the applicability of 2D-corr analysis, an extract of Habanero chili pepper (*Capsicum chinense* Jacq.) was used as a sample, since this food product has been widely studied by CoulArray sensors coupled with liquid chromatography [20,21]. In this extract, three characteristic peaks have been observed in all chromatograms, of which two correspond to capsaicin and dihydrocapsaicin (compounds that give the characteristic spiciness), while the composition of the other peak is not reported. Overall, the novelty of this work is providing a new tool based on chemometric analysis that allows a more reliable and faster evaluation of the purity of chromatographic peaks by CoulArray detectors. In addition, the practical importance of the study is that the chemometric tool can be in the future incorporated into commercial quality-control software packages, helping to improve quality control in the food industry.

## 2. Materials and Methods

### 2.1. Raw Material Source

The powders were obtained from Habanero peppers grown in a greenhouse in Sierra Papacal, Yucatán, Mexico (CIATEJ, Unidad Sureste). To obtain the powders, the peppers were dried in an oven at 65 °C for 72 h, milled with a mortar and pestle, then finally passed through a sieve with a mesh size of 500 µm.

### 2.2. Extraction of Chili Powder

About 500 mg of chili powder was mixed with 5 mL of solvent (80% MeOH, 20% H_2_O) in a falcon tube. The mixture was sonicated for 30 min at 25 °C and then centrifuged at 4700 rpm for 30 min. The supernatant was collected and filtered through a 45 µm PTFE filter and diluted 1:50 with mobile phase for analyzing by HPLC–ECD.

### 2.3. Chili Extract Analysis by HPLC–ECD

The chili extract analysis by HPLC–ECD was made with an Ultimate 3000 HPLC (Thermo Fischer Scientific, Waltham, MA, USA) system equipped with a membrane degasser, two peristaltic pumps, a sampling loop of 20 µL, and thermostat column compartment. The coulometric array detector (Thermo Fisher Scientific, Waltham, MA, USA) consisted of 16 porous graphite-working electrodes, poised at growing potentials from +100 to +850 mV. The chromatographic conditions were the same as in a previous work by Morozova et al. [20], specifically, the isocratic mobile phase containing water (55%) with 50 mM ammonium acetate at pH 4.4 with acetic acid and acetonitrile (45%) at a flow rate of 1 mL min^−1^.

### 2.4. Two-Dimensional Correlation Analysis

The two-dimensional correlation (2D-corr) analysis was conducted by using the software RStudio (version 1.4. 1717). The synchronous $\Phi \left(v_{1},v_{2}\right)$ and asynchronous $\Psi \left(v_{1},v_{2}\right)$ correlation chromatograms were calculated according to Noda’s theory [22]:

$$\Phi \left(v_{1},v_{2}\right)=\frac{1}{m-1}A^{T}A$$

$$\Psi \left(v_{1},v_{2}\right)=\frac{1}{m-1}A^{T}\mathrm{NA}$$

where $v_{1}$ and $v_{2}$ represent the time variable of the chromatogram. T refers to the usual matrix transpose operation. N is the Hilbert–Noda transformation matrix [22]:

$$N_{jk}\left\{\begin{array}{c} 0\\ \frac{1}{\pi \left(k-j\right)} \end{array}\right.\begin{array}{c} j=k\\ j\neq k \end{array}$$

In the present study, matrix A contains 16 chromatograms in rows (m = 16). Each row is a chromatogram at a specific potential (from +100 to +850 mV with a step of 50 mV).

### 2.5. Conditions for Chromatographic Separation of the First Peak

The chromatographic separation was made with an Ultimate 3000 HPLC system (Thermo Fischer Scientific) equipped with a column Accucore C18 (100 × 4.6 mm, 2.6 µm). The mobile phase consisted of: (A) water with ammonium acetate (50 mM) adjusted at pH 4.4 with acetic acid, and (B) acetonitrile 100%. The gradient used is presented in Table 1. To separate the compounds in the peak, the gradient began with a high percentage of the aqueous phase and a low flow; as time passed, the percentage of organic proportion increased along with the flow of the mobile phase.

### 2.6. Characterization of the Chromatographic Signals by HPLC–MS

The characterization of the chromatographic signals by HPLC–MS was made with an Ultimate 3000 HPLC system coupled with a high-resolution mass spectrometer Q Exactive Orbitrap. For full MS analysis, the ionization was made by electrospray ionization (ESI) in negative mode between a mass range of 50 to 750 *m*/*z* using the following conditions: sheath gas at 20, auxiliary gas at 10, auxiliary temperature 50 °C, spray voltage at +3.5 kV, capillary temperature at 320 °C, and RF S-lens at 65%. The ddMS^2^ measurements of the selected ions were performed with a resolution of 17,500 and AGC target at 5 × 10^5^. The peaks were analyzed with the software Compound Discover 3.1.0.305 (Thermo Fischer Scientific, Milano, Italy) to identify the chemical compounds.

## 3. Results and Discussion

### 3.1. Analysis by HPLC–ECD

**Figure 2:**
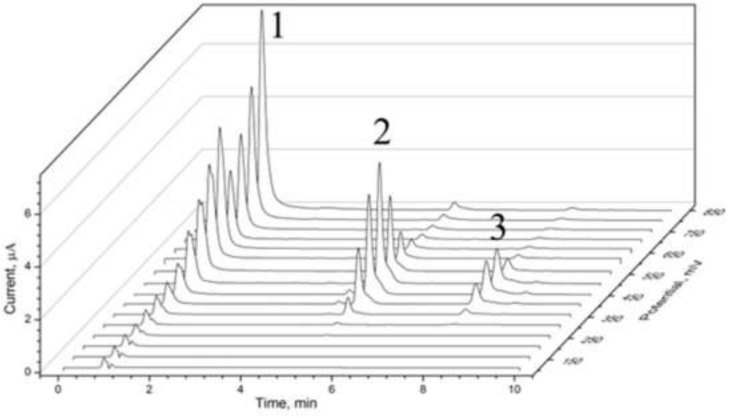
Current signal of the Habanero pepper extract from the 16 CoulArray channels poised at potentials from +100 to +850 mV with a step of 50 mV: (1) unknown peak; (2) capsaicin; (3) dihydrocapsaicin.

Figure 2 shows a representative chromatogram of a methanolic extract of Habanero chili pepper obtained from HPLC coupled with the CoulArray detector. The detector consists of an array of 16 independent electrochemical sensors, each poised at different and increasing potentials (from +100 to +850 mV vs. Pd/Pd+ pseudo-reference electrode, with a step of 50 mV). The chromatogram shows three main peaks: peaks 2 and 3 with a retention time of 5.17 and 7.74 min, respectively, were previously identified by Morozova et al. [20] and Oney et al. [21] as capsaicin and dihydrocapsaicin. However, peak 1, with a retention time of approximately 0.93 min, was not identified in the previous works and was considered an impurity. Therefore, these three peaks were further investigated using 2D-corr analysis.

**Figure 3:**
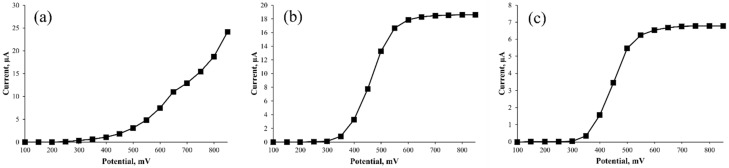
Corresponding hydrodynamic voltammograms of: (**a**) the first peak (unknown), (**b**) capsaicin, and (**c**) dihydrocapsaicin. Each point plotted in the graph corresponds to one area under the peak of one channel of the CoulArray detector.

The cumulative peak areas of each current signal as a function of the applied potential were evaluated on the three peaks. The resulting hydrodynamic voltammograms (Figure 3) define the characteristic redox behavior of the compounds associated with these three peaks. In particular, Figure 3a shows that the current signal increased with higher applied potentials, without showing any specific plateau value. The current started to raise at about +400 mV and continued to increase exponentially. Only a small shoulder was detected at about +650 mV. Although the absence of a well-defined plateau might indicate the presence of more than one molecule with a different potential of oxidation, the results from HPLC are not conclusive. For comparison, the hydrodynamic voltammograms of peaks 2 (Figure 3b) and 3 (Figure 3c) show a well-defined sigmoidal trend, with redox potentials of +350 and +550 mV, respectively. These results show that peaks 2 and 3 are formed only by a single molecule (or by more molecules with similar structure and redox potential behavior), i.e., capsaicin and dihydrocapsaicin, in agreement with previous findings [23,24].

In the next section, the purity of these peaks was verified by applying 2D-corr analysis.

### 3.2. Two-Dimensional Correlation Analysis

**Figure 4:**
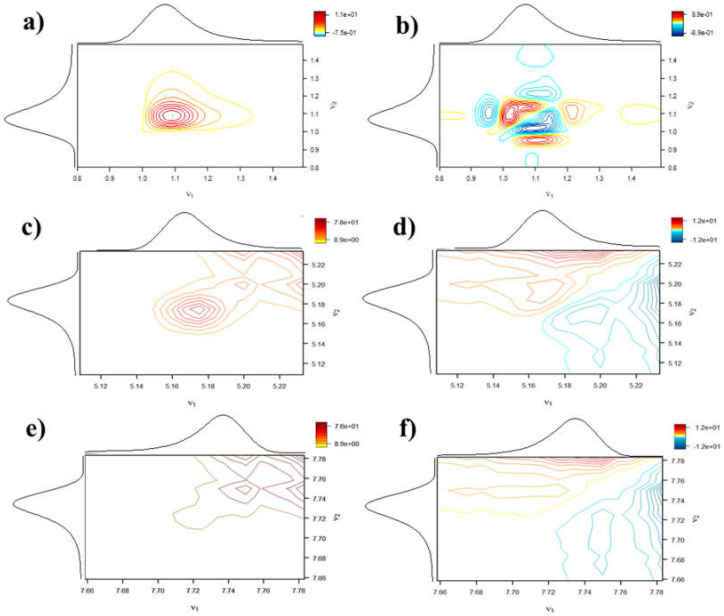
2D-corr analysis: (**a**) synchronous plot of peak 1, (**b**) asynchronous plot of peak 1, (**c**) synchronous plot of peak 2, (**d**) asynchronous plot of peak 2, (**e**) synchronous plot of peak 3, and (**f**) asynchronous plot of peak 3.

Figure 4 shows the results of the 2D-corr analysis of the chili extract with 16 coulometric sensors. In detail, Figure 4a,c,e show the result of the synchronous 2D-corr analysis of the first three peaks highlighted in Figure 2. Although these graphs do not give information on the composition of the peaks, they can provide a way to combine the information contained by the 16 signals in a single comprehensive chromatogram. This is the first important result, which solves the common challenge of displaying the results of several chromatograms in a simpler way. This achievement is mainly due to the capacity of synchronous maps to represent only the similarities or differences between the chromatographic peaks occurring during the elution of the peaks.

Similarly, Figure 4b,d,f show the resulting asynchronous 2D-corr analysis—the first three peaks highlighted in Figure 2, respectively. In detail, Figure 4b shows six cross-peaks: three of them are positive and the others negative. These peaks are asymmetric considering the main diagonal line, which is crossing the graph. The number of asymmetric peaks expresses the number of components that behave differently according to the changes in potential. Accordingly, these six signals (three on each side of the diagonal line) indicate that peak 1 of Figure 2 is composed of at least three independent antioxidant species, each having a different redox behavior. Overall, the different redox ability of these compounds allows us to detect their presence despite having the same retention time.

On the other hand, Figure 4d,f correspond, respectively, to peak 2 (capsaicin) and 3 (dihydrocapsaicin). These maps do not reveal multiple signals but support the hypothesis that only one single compound is represented by these peaks. This confirms the result observed at the hydrodynamic voltammograms and previous analysis.

Overall, the 2D-corr analysis revealed that peak 1 was not a single substance but could be a mixture of more than one analytical species, whereas peaks 2 and 3 correspond only to one single compound, i.e., capsaicin and dihydrocapsaicin, respectively, as reported previously [23,24].

The next section will verify the presence of other compounds in peak 1 by using high-performance liquid chromatography coupled with a high-resolution mass spectrometer.

### 3.3. Identification of Peaks by HPLC–MS

**Figure 5:**
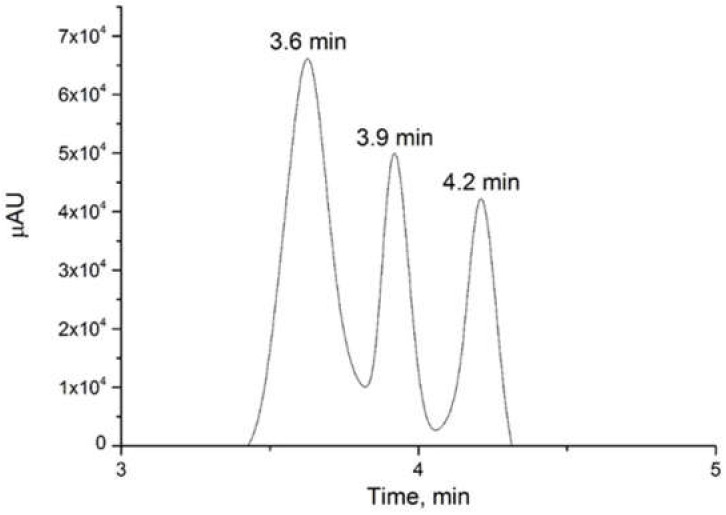
Chromatogram of a chili extract by UV–VIS detector at 280 nm obtained with the optimized gradient conditions (Table 1).

Peak 1, with a retention time of 0.93 min, was separated from the conditions previously mentioned in the methodology and the gradient described in Table 1. This was possible mainly to the change of the mobile phase conditions. The modified gradient started with 95% water with ammonium acetate 50 mM (phase A, pH 4.4) and resulted in a higher retention of the molecules with the stationary phase and increased the performance of the column. The initial condition was maintained for 5 min, then mobile phase B (acetonitrile) was increased to 100% for the elution of the compounds that have a higher retention time (capsaicinoids). These conditions were maintained for 14 min, with a final return to the initial conditions. With an improved gradient due to the decrease in the eluting force of the mobile phase, it was possible to observe in the chromatogram three separate peaks with the retention times corresponding to 3.6, 3.9, and 4.2 min (Appendix A). The separation of the peak into three different compounds (Figure 5) confirmed the result obtained by the 2D-corr analysis, demonstrating the possibility of the presence of three species in peak 1.

After the separation of the peaks, three corresponding compounds were identified using HPLC coupled with a MS detector (Table 2) in ESI negative ionization mode. The three detected masses corresponded to quinic acid, ascorbic acid, and phenylalanine, according to their exact measured mass and comparison with the independently measured standards (Appendix B).

The three identified compounds are considered metabolites present in different kinds of peppers [23]. Ascorbic acid is a water-soluble vitamin that has been reported in Habanero pepper in concentrations of 43 to 247 mg per 100 g of fresh chili, which corresponds to values between 50 and 100% of the daily requirement [24]. On the other hand, quinic acid is an organic acid considered to be one of the main organic acids present in chili peppers, together with citric acid and malic acid [25]. On the other hand, phenylalanine is an amino acid that has been found in the fruits of Chinese capsicum. It plays an important role as a precursor in the biosynthesis of capsaicinoids [26]. It has been reported by Castro-Concha et al. [27] that its concentration in the Habanero pepper is low in the early stages of the development of the fruit (2.5 nmol g^−1^) and increases significantly as the fruit ripens, reaching a concentration of approximately 22 nmol g^−1^.

## 4. Conclusions

Two-dimensional correlation analysis using synchronous and asynchronous plots was applied to determine the peak purity in the analyzed chili extract by HPLC coupled with a coulometric array detector. The synchronous plot provided information on the number of complex peaks present in the chromatogram, while the asynchronous plot was useful to assess the purity of peaks. The asynchronous plot showed the sequential changes of the signal due to the change of potential during each time period and it was possible to observe the number of compounds in each chromatographic peak. The application of this approach to the analysis of an unresolved peak of Capsicum chili extracts demonstrated the presence of three different compounds. These compounds were consequently separated and identified by high-resolution MS analysis as phenylalanine, quinic acid, and ascorbic acid. The results showed the potential of two-dimensional analysis with an array of coulometric sensors to verify peak purity quickly and reliably. This will help to avoid quality-control errors and thus ensure that consumers are protected from possible product adulteration or contaminations. Moreover, 2D-corr analysis should be incorporated into commercial quality-control and statistical software packages.

## Figures and Tables

**Table 1:** Gradient used for chromatographic separation of the first peak.

Time (Minutes)	Phase A (%)	Phase B (%)	Flow (mL min^−1^)
0	95	5	0.2
5	95	5	0.3
6	0	100	0.6
20	0	100	0.6
22	95	5	0.2
35	95	5	0.2

**Table 2:** Molecules identified in the chili extract by high-resolution mass spectrometry in negative ionization mode.

Molecule	Formula	Retention Time (Minutes)	Molecular Weight	Theoretical *m*/*z*	Measured *m*/*z*
Quinic acid	C_7_H_12_O_6_	3.593	192.06369	191.05611	191.05646
Ascorbic acid	C_6_H_8_O_6_	3.943	176.12410	175.02481	175.02480
Phenylalanine	C_9_H_11_NO_2_	4.229	165.07933	164.07170	164.07203

**Figure A1:**
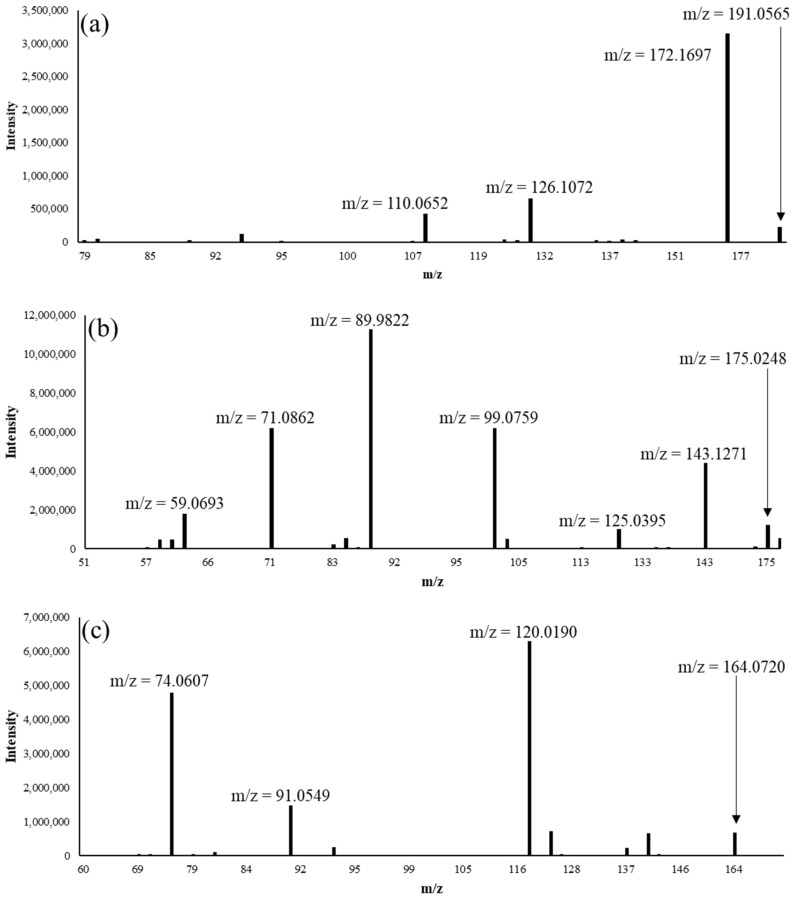
Fragmentation spectra of (**a**) quinic acid, (**b**) ascorbic acid, and (**c**) phenylalanine obtained by HRMS.

## Data Availability

Data is available from the authors on request.

## Appendix A

**Table A1:** Chromatographic parameters associated with the peaks.

Compound	tr	k′	N	H
Quinic acid	3.59	6.98	307	0.33
Ascorbic acid	3.94	7.76	1480	0.07
Phenylalanine	4.23	8.40	1192	0.08

Note: tr: retention time, k′: capacity factor, N: number of theoretical plates, H: theoretical plate height.

**Table A2:** Chromatographic parameters associated with the separation between the peaks.

	Selectivity Factor (α)	Resolution Factor (Rs)
Quinic acid–ascorbic acid	1.11	0.57
Ascorbic acid–phenylalanine	1.08	0.64
